# A Review on the Progress in Core‐Spun Yarns (CSYs) Based Textile TENGs for Real‐Time Energy Generation, Capture and Sensing

**DOI:** 10.1002/advs.202304232

**Published:** 2023-08-21

**Authors:** Akshaya Kumar Aliyana, George Stylios

**Affiliations:** ^1^ Research Institute for Flexible Materials School of Textiles and Design Heriot‐Watt University Edinburgh EH14 4AS UK

**Keywords:** core spun yarns (CSYs), energy harvesting and storing, internet of things (IoT), smart sensing devices, stribomaterials, textile triboelectric nanogenerators (T‐TENGs)

## Abstract

This review is a critical analysis of the current state‐of‐the‐art in core spun yarn textile triboelectric nanogenerators (CSY‐T‐TENGs) for self‐powered smart sensing applications. The rapid expansion of wireless communication, flexible conductive materials, and wearable electronics over the last ten years is now demanding autonomous energy, which has created a new research space in the field of wearable T‐TENGs. Current research is exploring T‐TENGs made from CSYs as stable and reliable energy harvesters and sensing devices for modern wearable IoT platforms. CSY‐TENGs are emerging as an important technology due to its simple structure, low cost, and excellent performance in converting mechanical energy into electrical energy and due to its sensing ability. This paper provides a critical review on current progress, it analyzes the unique advantages of CSYs T‐TENGs over conventional T‐TENGs, it describes fabrication techniques and discusses the materials used along with their properties and electrical performance characteristics, and it highlights the recent advancements in their integration with self‐excitation circuits, charge storage devices and IoT‐enabled smart sensing applications, such as environmental and health monitoring. In the conclusion, it discusses the challenges and future directions of CSYs T‐TENGs and it provides a future road map for optimization, upscaling, and commercialization of the technology.

## Graphical abstract

This paper provides a critical review on current progress, it analyzes the unique advantages of CSYs T‐TENGs over conventional T‐TENGs, it describes fabrication techniques and discusses the materials used along with their properties and electrical performance characteristics, and it highlights the recent advancements in the integration of CSYs T‐TENGs with self‐excitation circuits, charge storage devices and IoT‐enabled smart sensing applications.

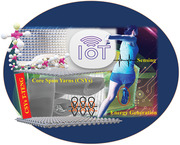

## 1 Introduction

The use of fossil fuels is affecting the global climate and human health.^[^ ^1^, ^2^, ^3^, ^4^ ^]^ According to Harvard University's research, coal and diesel are responsible for air pollution, killing seven million people a year, which is one in five deaths worldwide.^[^ ^5^ ^]^ To diminish carbondioxide emissions and air pollution, there is a need to rapidly shift toward low‐carbon sources and renewable energy technologies.^[^ ^6^, ^7^ ^]^ Renewable energy will play a key role in the decarbonization of world energy systems in the forthcoming decades. According to a recent report by the International Energy Agency (IEA), electricity generation from hydropower, wind, and solar is predicted to increase but will only be able to meet half of the projected increase in global electricity demand.^[^ ^8^ ^]^ Hence the rapid production of renewables and the development of new energy technologies are in ever‐increasing demand and Nanogenerators (NGs) in the broad sense are set to become the next leading technology in the field of renewable energy, which converts mechanical and/or thermal energy into electricity.^[^ ^9^ ^]^ NGs are classified into three typical methodologies: piezoelectric, triboelectric, and pyroelectric NGs.^[^ ^10^, ^11^, ^12^, ^13^ ^]^ Most of the research is diverted toward piezoelectric and triboelectric NGs, with both being able to convert mechanical energy into electrical energy more efficiently.^[^ ^14^ ^]^ The need for autonomous energy “without the use of batteries” for the Internet of Things (IoT) for human‐machine interfacing, Artificial Intelligence (AI) and Machine Learning (ML) is focusing research interest in wearable Triboelectric Nanogenerators (TENGs) due to being lightweight, flexible, cost‐efficient, simple in design and structure, and biocompatible.^[^ ^15^, ^16^, ^17^, ^18^ ^]^ Materials scientist Zhong Lin Wang of the Georgia Institute of Technology founded TENGs and reported the first device in 2012.^[^ ^19^ ^]^ The science of TENGs is based on the coupling of the triboelectric effect and electrostatic induction to generate electricity from mechanical friction and displacement. According to device design, type and choice of materials, TENGs belong to the green energy domain with diverse application capabilities in human motion, vibration, sliding or rolling, waterdrops falling or raining, vehicle motion, water waves in the sea and river, wind energy etc.^[^ ^20^, ^21^, ^22^, ^23^ ^]^ The combination of wearable monitoring with autonomous TENG energy, demands the advances of smart textiles in order to achieve self‐powered sensing capabilities^[^ ^24^, ^25^ ^]^ and wireless data communications (IoT).^[^ ^26^ ^]^ Smart textiles have unique features such as being capable of large curvatures that enable the wrapping of the human body with comfort, good resistance to fatigue during wear, softness, breathability, washability, and permeability, making them ideal platforms for wearable monitoring, and for fabricating flexible and wearable TENGs. In order to fully develop this technology however some challenges need to be addressed for improving their performance. T‐TENGs usually have lower output compared to traditional TENGs due to lower triboelectric charge density of the textile materials used and lower contact surface area between the fabrics.^[^ ^27^ ^]^ The design of T‐TENGs is dependent on the textile structure and on the integration of multiple layers of fabrics and electrodes to realize the T‐TENGs device, which are being responsible for the lower efficiency and reliability of the device. Their fabrication process is also challenging, requiring precise alignment, and bonding of the different fabric layers, and it also poses durability issues due to the wear and tear of the textile materials, especially when subjected to repeated mechanical stress, abrasion or washing. This can affect the performance and lifespan of the device. T‐TENGs are less environmentally stable than traditional TENGs due to the sensitivity of the textile materials to humidity and temperature changes, which can affect the reliability and reproducibility of the device under different environmental conditions, and can limit their practical applications.^[^ ^28^ ^]^

For these reasons Core Spun/Sheath Yarns (CSYs) based T‐TENGs are being researched, and exploratory trials have revealed interesting results in output, underpinning their unique advantages.^[^ ^29^ ^]^ CSYs based T‐TENGs are a better approach for integrating into complete fabrics, they consist of an inner conductive electrode layer and a further outer layer covered with bundles of twisted microfibers/nanofibers or with coated triboelectric layers. Relatively few efforts have been reported to develop CSYs TENGs using different textile structures by knitting, weaving or braiding.^[^ ^30^, ^31^, ^32^, ^33^ ^]^ Despite of their fabrication stability and adaptability for wearable garments being promising, all reported studies are limited in producing the required power density to run wearable sensors, and there are difficulties for implementing the necessary Power Management Circuits (PMCs) into fabrics.

**Figure 1:**
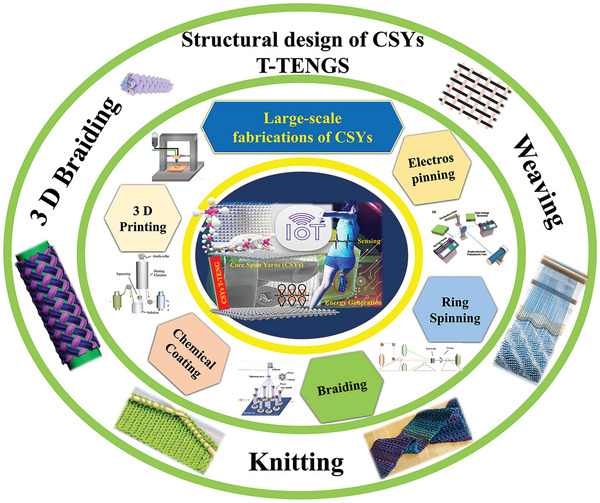
A schematic overview illustration of this review paper showing various large‐scale fabrications, structural CSY design techniques and production processes of CSYs T‐TENGs textile layers.

For successful wearable self‐powered smart sensing, optimized device fabrication and integration of CSYs T‐TENGs with PMCs are vital for wearable applications. Looking at the literature a review paper in 2020 by Rayan et.al^[^ ^34^ ^]^ proposed a general categorization of fiber based T‐TENGs and Qian Chen et. al provided an in‐depth discussion on fabrication of tribo material technical yarns in 2022.^[^ ^35^ ^]^ And despite the increased interest and recent progress in T‐ TENGs, there is lack of any extensive analytical discussion focus on CSYs based T ‐TENGs. Aspects of structural design and fabrication of fabric designs, layer integration, self‐charge extraction, miniaturized PMCs, energy storage and charge regulations circuits are all important research areas for discussion. In consequence this review presents a detailed, focused, and critical, exploration of the recent research progress and development strategies of CSYs T‐TENGs to help overcoming its challenges and achieving integration and use with IoTs wearable devices and beyond. **Figure** 1 is a schematic illustration of the areas covered in this review paper. The discussion topics start with an insight into numerous large‐scale fabrication and structural design CSY techniques, followed by a critical analysis on self‐charge extraction, supercapacitors, and PMCs to help enhancing the CSYs T‐TENGs electrical output performance. It then goes on to discuss CSYs T‐TENGs as sensing devices, along with their adaptability of wireless data transmission for wearable IoT devices. Finally, a research road map is provided with recommendations to solving present challenges which pave the way to future prospectives of CSYs T‐TENG for wearable self‐powered sensing application platforms.

### 1.1 Working Principles of T‐TENGs

The energy harvesting process of a TENG is based on the triboelectric and electrostatic effects of at least two materials as they come in contact with each other.^[^ ^19^ ^]^ Triboelectric charging is a well‐identified phenomenon of contact electrification in which specific materials become electrically charged after they are separated from another polarity material with which they were in contact.^[^ ^35^, ^36^ ^]^ Further, the triboelectric effect activates an electrostatic charge build up on the surface of the two conducting electrodes due to contact separation, which drives the flow of electrons between the conducting electrodes and generates Alternating Current (AC).

**Figure 2:**
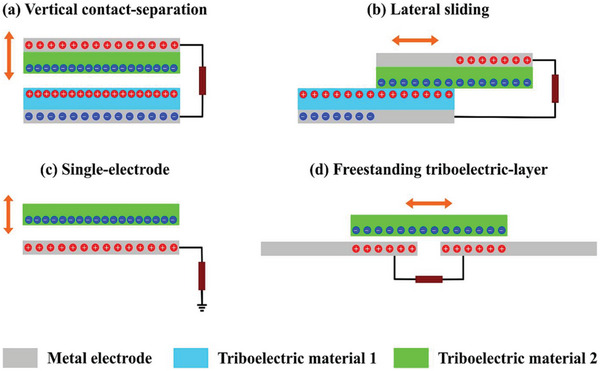
Schematic illustrations of four working modes of TENGs. a) Contact‐Separation (C‐S) mode, b) Lateral‐Sliding mode (L‐S), c) Single‐Electrode mode (S‐E), and d) Free‐Standing (F‐S) mode. Reproduced with permission.^[^ ^37^ ^]^ Copyright 2021, Elsevier.

The researcher community has considered four different types of TENG working modes and divided them into: Contact Separation mode(C‐S), Lateral Sliding mode (L‐S), Single Electrode mode (S‐E), and Free‐Standing mode (F‐S). **Figure** **2** states the four different types of the working mechanism of traditional TENGs. Their working mechanism is equally applicable to the CSYs T‐TENGs as well. The term CSYs means Core Spun/Sheath Yarns, which is a type of yarn in which a central core of either synthetic or natural fibers yarn is metal platted with conducting material(s), or it consists of a single conducting metal yarn, and which has a sheath of tribo electric material wrapped around its core, usually from micro/nanofibres The conductive cores of the CSYs act as electrodes and the spun/sheath layers as the tribo layers. The charges generated on the tribo layers are transferred to the electrodes, creating a potential difference between them, and are generating electrical power.

**Figure 3:**
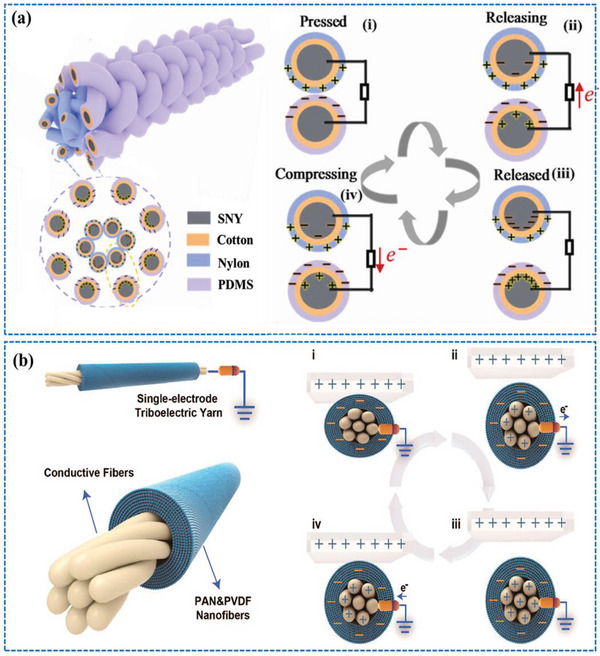
The working mechanism of CSYs T‐TENGs in (a) vertical Contact Separation (C‐S) and (b) Single‐Electrode (SE) configurations. a) Reproduced with permission.^[^ ^30^ ^]^ Copyright 2021, Elsevier b) Reproduced with permission^[^ ^38^ ^]^ Copyright 2020, American Chemical Society.

Contact separation (C‐S) is the most studied TENG configuration, but practically less applicable in CSYs T‐TENGs, which consist of two electrodes composed of two different charge polarity textile‐based materials, being built around metal electrodes. The L‐S mode is the most interesting TENG configuration with regard the CSYs T‐TENGs due to its suitability for collecting mechanical energy from the in‐plane movement of textiles as it utilises the normal wearing movement changes of fabrics in day today life activities. The S‐E mode comprises of an electrode embedded with either a positive or negative charge polarity textile material, when a moving object encounters the single electrode structure, a potential difference is formed between the electrode and the ground (zero potential), and the electrons flow between the two to form an electric current. For wearable TENGs the S‐E configuration is the most investigated wide‐ranging configuration, which is also considered with skin as the positive tribo layer. The F‐S is further developed from single electrode TENG configurations. In the L‐S mode there is no need for grounding, so all electrodes can move freely.^[^ ^38^ ^]^ The basic working principle and the performance of the C‐S and S‐E mode based CSYs T‐TENG is represented in **Figure** **3**. It must be said that all four working modes have different characteristics which are being used to their maximum output depending on the natural frequency and force generation during testing.

### 1.2 CSYs T‐TENGs Materials

Over the last 3 years the direction of T‐TENGs device development is towards CSYs T‐TENGs because of core electrode connectivity throughout the device and multivariant material properties of the wrapped materials which is the result of integrating fibers, yarns, and fabrics with functional components. A common strategy, that is being used for improving the electrical performance of T‐TENGs is coating of functional materials onto textile surfaces by using dip, pad, solgel, spray coating, chemical vapour deposition (CVD), plasma, electrochemical and electrostatic coatings, layer by layer ions assembly, drop casting, printings, etc.^[^ ^39^, ^40^, ^41^ ^]^ However, these methods have limitations in uniformity, control, cost, complexity, durability, washing, flexibility, breathability, adhesion, and poor scale‐up productivity. At the same time most, electrodes used in traditional T‐TENGs are conductive metal wires or sheets on the top of the tribo layers, which have lower contact with the tribo layers, resulting in poor adsorption, as well as losing wear comfort. The need for real time implementation of wearable T‐TENGs demands the fabrication of CSYs with tribo materials. T‐TENGs may always vary with material suitability and adaptability for every textile fabrication, depending on the wearing comfort of the fabric.

**Figure 4:**
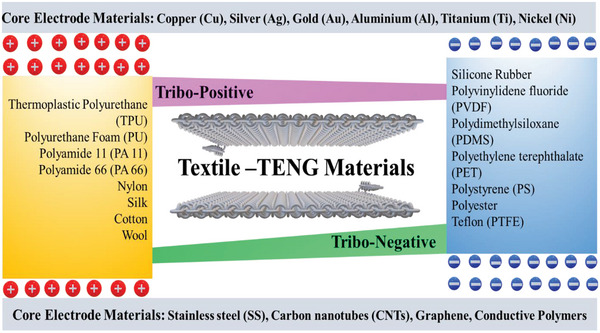
List of presently used textile and core electrode materials for CSYs T‐TENGs.

**Figure** **4** considers the more suitable textile tribo and core electrode materials for novel CSYs T‐TENGs fabrication. Their basic properties are determined from their core part, fiber type; their fiber length‐to‐width ratio, uniformity, strength, flexibility, extensibility, elasticity, cohesiveness, bending, shear, compression ability and surface roughness. The sheath materials are also designated reliant on their triboelectric properties which are compatible for fabric design, wear, and use. Figure 4 depicts the textile tribo materials on both ends, which have positive or negative polarities depending on their electron donating/accepting affinity, along with a list of suitable core electrode materials for CSYs T‐TENGs. The conductive core materials used in CSYs T‐TENGs can be metals and conducting polymers, such as Copper (Cu), Silver (Ag), Polyethylene Oxide (PEO) doped with Carbon nanotubes (CNT), other metals like Stainless Steel (S‐S), Aluminium (Al), Nickel (Ni), Gold (Au) and Titanium (Ti). The conductive core provides the pathway for the flow of electrons generated by the triboelectric effect and is having much less resistance value per meter (<500 Ω/m). The sheath fibers are from natural or synthetic materials, such as cotton, polyester, nylon, silk, wool etc., they protect the conductive core from damage and help to maintain the inner separation between the two layers of CSYs, they also sometimes enable dual TENG configurations. These dielectric materials are used to generate a barrier between the conductive core and the insulating fibers, which helps to prevent leakage of the charge generated by the triboelectric effect. Materials such as Polyethylene terephthalate (PET), Polytetrafluoroethylene (PTFE), Polyurethane (PU), Polyvinylidene fluoride (PVDF) and Polydimethylsiloxane (PDMS) are commonly used as dielectric materials. Overall, the materials used in CSYs T‐TENGs are carefully chosen to provide the necessary electrical and mechanical properties for efficient energy harvesting.

## 2 Large‐Scale Fabrications of CSYs

The latest research efforts are towards developing more realistic T‐TENGs layers. CSYs T‐TENGs devices of this category are structurally and practically well matching with either single‐electrode or lateral sliding working mode. Since 2021 exciting progress has been made in developing CSYs by using various lab‐based small scale fabrication techniques, which show promising application prospects for wearable end uses. However, the lack of simple, efficient, and large‐scale manufacturing technologies has greatly hindered the fabrication and practical uses of CSYs‐T TENGs. Here we summarize the existing preparation methods starting from electrospinning, ring spinning/double spinning and small‐scale braiding to 3D printing and also discussing various chemical coating methods.

Electrospinning is a process used to create ultrafine fibers with diameters ranging from a few nanometres (nm) to several micrometers (µm). This process involves the use of a high electric field to draw a liquid polymer solution or melt into micro or nanofibers, which is then solidified by various means such as evaporation or chemical crosslinking.^[^ ^42^, ^43^ ^]^ The basic method of electrospinning has remained mostly unaltered since its conception in the 1600s.^[^ ^44^ ^]^

**Figure 5:**
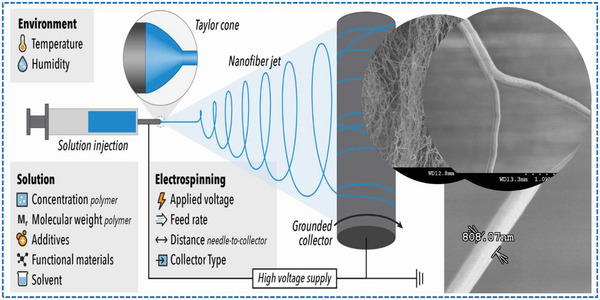
Schematic illustration of electrospinning of nanofibers and its regulatory parameters. Reproduced with permission.^[^ ^45^ ^]^ Copyright 2022, Elsevier.

Electrospun nanofibers have several prominent properties that make them more suitable for realizing the high output performance needed for wearable TENGs applications, because they have a very high surface area‐to‐volume ratio due to their nanoscale fiber structures. Their porous structure and small diameter, makes nanofibers unique for highly demanding applications.^[^ ^46^ ^]^ Despite their small size, nanofibers can have high tensile strength, which can substantially enhance the effective frictional contact area and breathability, and hence improving the overall performance of CSYs. Electrospinning is also used for alignment of nanofibers, nanofillers such as nanotubes and nanowires inside electrospun layers.^[^ ^22^, ^47^ ^]^ **Figure** **5** illustrates a typical electrospinning process, listing the conditions that affect nanofiber properties. The electrospinning process starts with the synthesis of a polymer solution, which is loaded into a pumping syringe connected via a tube to a needle like spinneret. An electric field is applied to the polymer solution using a high‐voltage power supply. The electric field creates an electrostatic force that draws the polymer solution through the small opening of the spinneret. As the polymer solution is drawn through the electric field, it forms fine fibers that are collected on a grounded collector. Academics have investigated various type of grounded collection systems, which include, stationary platforms, rotary/movable drums/plates/disks. G. Stylios et.al invented a new electrocarding system, which facilitates the orderly collection of nanofibres into a fibre web bundle and their conversion into nanosliver by fibre drawing, prior to yarn spinning and fabric weaving.^[^ ^48^, ^49^ ^]^

**Figure 6:**
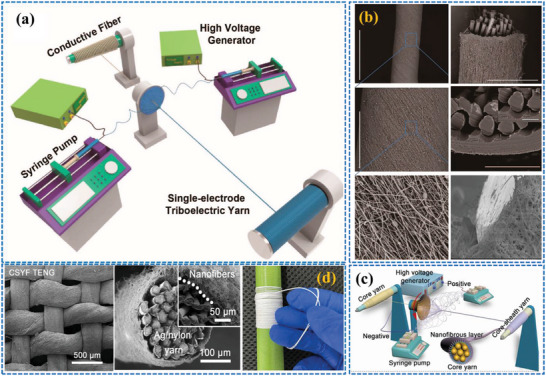
a) Production process of single electrode triboelectric yarn using a double spinning technique, b) cross sectional and surface view of the CSYs, c) schematic illustrations of double spinning yarning system and d) SEM images of core spun nanoyarns and woven fabrics. a,b) Reproduced with permission.^[^ ^38^ ^]^ Copyright 2020, American Chemical Society. c,d) Reproduced with permission.^[^ ^55^ ^]^ Copyright 2022, Elsevier.

The parameters of the electrospinning process, such as solution concentration, flow rate, collector type, tip‐to‐collector distance, and electric field strength, are adjusted to control the size and morphology of the resulting nanofibers (fiber diameter, morphology, orientation, and alignment).^[^ ^50^, ^51^ ^]^ To fabricate nanofibers to nanoyarns, a yarn double spinning method can be used^[^ ^52^ ^]^ enabling various multidisciplinary application.^[^ ^51^, ^53^ ^]^ The double spinning and continuous yarning system produces strong core nano yarn with tuneable yarn surface properties,^[^ ^54^ ^]^ **Figure** **6** shows a laboratory scale production process of core spun nanoyarns using the double spinning system.

**Figure 7:**
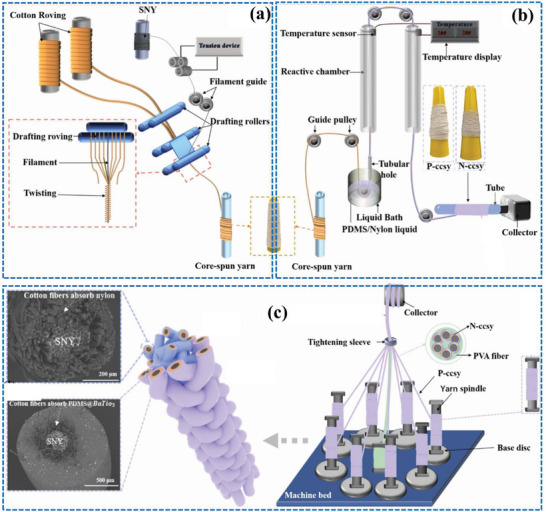
Schematic illustration of multivariant tribo material layer fabrications, a) ring spinning method, b) chemical coating method and c) 3D braiding method. Reproduced with permission.^[^ ^30^ ^]^ Copyright 2021, Elsevier.

The winding speed of the nanofibers over the core yarns are important to create uniform and parallel nanofibers over the conducting core yarn. Gao et. al extended CSYs T‐TENG studies through CSYs with a hierarchical yarn architecture. They made use of silver‐plated nylon yarns (SNYs) as core and insulating cotton fibers as sheath layers, where SNYs serve as highly conducting electrode and cotton fibers serve as base materials for absorbing/coating the triboelectric core materials. The fabricated yarns show improved strength, enhanced electrical conductivity, and increased flexibility compared to conventional yarns. The production process of core‐spun nanoyarns using the double spinning and yarning system however is more intricate and time‐consuming and requires specialized equipment and skilled operators, making it unsuitable for mass production. Achieving stable and uniform nanofiber distribution within the CSYs structure is challenging and here the choice of the core material is crucial, to overcome poor adhesion between the core yarn and the nanofibers, which is critical for the overall integrity and durability of the core spun yarn, because weak adhesion leads to reduced mechanical strength and durability, and compromised performance.^[^ ^56^, ^57^ ^]^ Despite these obstacles, ongoing research is trying to defeat these challenges and optimize the production process and scalability. **Figure** **7** illustrates the continuous manufacturing processes of CSYs using multivariant methods, which include ring spinning, chemical coating, and braiding techniques. Ring spinning is used for producing CSYs with the coating apparatus to make negative and positive tribo material based CSYs.^[^ ^30^ ^]^ Braiding can be used to produce CSYs using several different techniques.

The round braiding technique involves braiding multiple fibers around a central core, the central core would be the conductive wire or fiber used to collect the electrical charges generated by the T‐ TENGs. The flat braiding method comprises of braiding multiple fibers in a flat pattern, creating a ribbon‐like structure. Tubular braiding, is used by braiding multiple fibers in a tubular pattern, creating a hollow structure. The choice of braiding technique will depend on the specific requirements of the CSYs T‐TENG application. For example, round braiding may be preferred for applications where a high degree of tensile strength is needed, while tubular braiding may be ideal for applications where flexibility and conformability are more critical. The number of fibers used in the braiding process influence the diameter and properties of the CSYs. To overcome the limitations of complex processing of double electrospinning, Yingying Li et. al reported a large‐scale method to fabricate CSYs using braided fibers. These braided fibers maintain uniform thickness and are of continuous length, and are achieved by braiding PVDF around silver‐coated nylon yarns.^[^ ^58^ ^]^

Three‐dimensional (3D) printing is another practical method for the manufacture of CSYs. This process involves depositing a polymer or composite material layer by layer, allowing for the creation of complex structures with precise control over the dimensions and properties of the resulting CSYs. 3D printing of CSYs involves printing a core filament of conductive material, such as copper wire or conductive polymer, and then depositing the polymer or composite material around the core filament in a continuous pattern. The resulting CSYs are used as the active component in a T‐TENG device. Alternative approaches involve printing a matrix material with embedded conductive fibers, such as carbon nanotubes (CNTs) or metal fibers. The matrix material provides the structural support for the CSYs, while the conductive fibers serve as charge carriers. The advantages of using 3D printing for CSYs production include the ability to create complex structures with precise control over the dimensions and properties of the resulting CSYs as well as the ability to easily incorporate additional components, such as sensors and printable small‐scale devices into the CSYs. Challenges still exist however in optimizing the printing parameters and the composition of the combined materials, as well as in scaling up the production of 3D printed CSYs for industrial applications.

**Figure 8:**
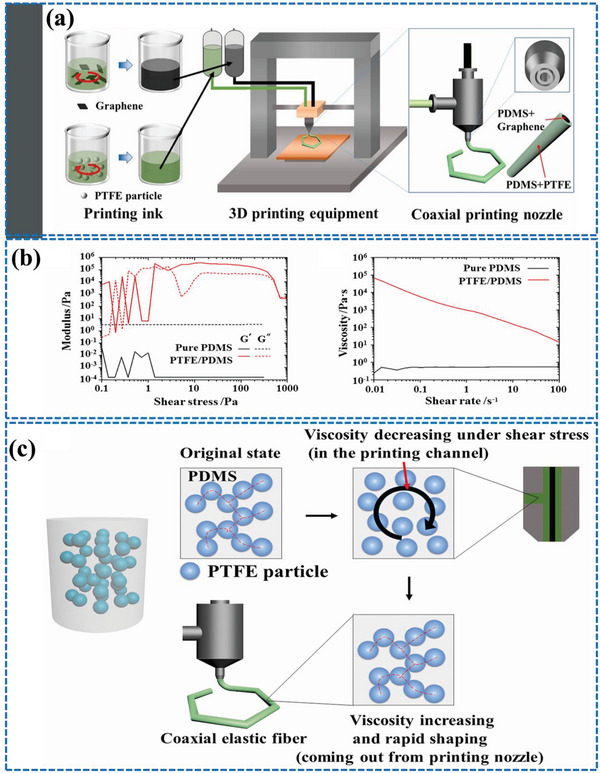
a) Schematic illustrations of 3D printing technology used for CSYs production. b) relationship of storage modulus, loss modulus and viscosity growth of pure PDMS prepolymer and PTFE filled PDMS prepolymer under shear stress c) structure evolution of the PDMS prepolymer filled with PTFE particles under shear stress. Reproduced with permission.^[^ ^59^ ^]^ Copyright 2021, Elsevier.

**Figure 9:**
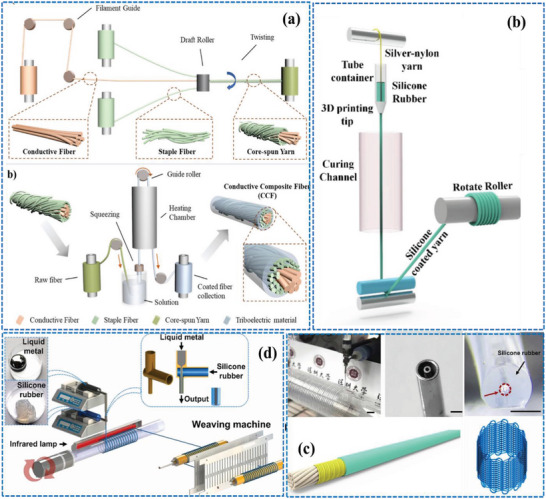
Schematic illustrations of the production process of CSYs using a) combined yarn spinning and chemical coating, b) passing conductive silicone filament through curing channel, c) cross‐sectional and surface view of fabricated CSYs and d) schematic illustrations of continuous process of double spinning and weaving using liquid alloys and silicone materials. a) Reproduced with permission.^[^ ^63^ ^]^ Copyright 2020, American Chemical Society. b,d) Reproduced with permission.^[^ ^64^ ^]^ Copyright 2022, Elsevier. c) Reproduced with permission.^[^ ^65^ ^]^ Copyright 2022, Elsevier.

**Figure** **8** depicts the CSYs production process using 3D printing techniques. Graphene and PDMS are used as printing materials which are converted from newtonian to non‐newtonian type to meet rapid prototyping requirements of 3D printing. Usually, Graphene and PTFE are used as fillers in PDMS and contribute to continuous stretchable smart fibers by 3D printing, where material viscosity plays a crucial role during the printing process.^[^ ^59^, ^60^ ^]^ There are a set of chemical coating methods reported for developing CSYs with different properties and functionalities. Chemical coating modifies the properties of the CSYs to achieve the desired performance characteristics, such as increased conductivity, durability, or sensitivity to specific environmental stimuli, depending on specific application requirements.^[^ ^61^ ^]^ The most investigated chemical coating methods for CSYs include, electroplating, chemical vapor deposition (CVD), surface modification and sol‐gel coating. The choice of coating method will depend on the demands of the CSYs T‐ TENGs application, as well as the properties of the CSYs and the coating material. The chemical coating process can also combine a core‐spun spinning method and a surface coating. A triboelectric material is then further coated over the surface of the CSYs. This method is suitable for various conductive core wires (such as silver yarn, copper wire and stainless‐steel wires), short fibers (Cotton, Silk, Polypropylene), and triboelectric materials such as PDMS, TPU, Nylon, PVC, and PVDF. Here existing technologies are divided into three categories: spraying triboelectric materials on metal wires, adding conductive electrodes after spinning a tubular triboelectric layer, and coaxial spinning. **Figure** **9** illustrates the fabrication method of both yarn spinning and chemical coating of conductive composite fibers. Most CSYs are usually prepared by coating a dielectric material on a conductive material, but those preparation processes are complex and decrease the breathability, washability, and air permeability of the CSY fabrics. To solve these problems, Chuan Ning et. al investigated one‐step coaxial wet spinning. Here they used 0.18 mm fibre diameter consisting of a liquid metal (LM) core and a polyurethane (PU) sheath. Due to good mechanical properties between the materials, there is no interface incompatibility of the triboelectric fibers.^[^ ^62^ ^]^

**Figure 10:**
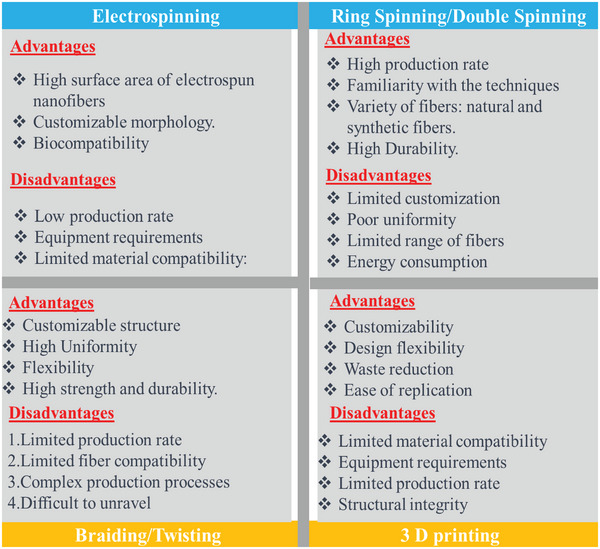
Comparison table of the major techniques used for CSY fabrication.

Yuying Cao et. al explored a chemical coating method to develop a conductive silicone filament structure. Figure 9b describes the continuous fabrication of CSYs, which consists of a tube length of 8.5 cm, with a cone‐shaped nozzle of 1 mm used as a squeegee to load the silicone precursor to the conductive yarn. The coated yarn was subsequently pulled out from the squeegee, traveling through a heating chamber (100^◦^C), where the silicone was cured.^[^ ^64^, ^66^ ^]^ This technique created a wrapped coaxial structure for the CSYs and has good flexibility and elasticity properties. **Figure** **10** depicts the advantage and disadvantages of the major production techniques used for CSYs.

## 3 Structural Design of CSYs‐TENGs Layers and Output Performance

**Figure 11:**
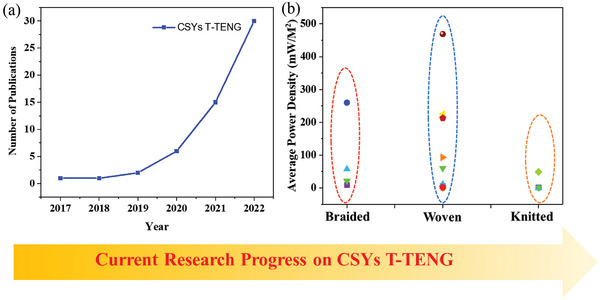
a) Trends showing the number of publications specifically related to CSYs T‐TENGS and b) statistical data representation of average power density with respect to different CSYs sample structures (Braiding, Woven and Knitting). The data was obtained from Google Scholar using the keywords “core spun yarns‐based T‐TENGs”. All searches were performed in April 2023.

The structural design of CSY fabric layers for T‐TENGs is critical to achieving the desired performance characteristics. The structural design affects the flexibility, tensile strength, CSYs density, washability, wearability, and other properties of the CSYs layers, which in turn can impact on the output performance of the T‐ TENGs. Novel designed CSYs layers can maximize performance and improve suitability for a wide range of smart textile applications.^[^ ^67^, ^68^ ^]^ To improve output performance, researchers combined T‐TENGs with new variations of CSYs‐based fabric layers and core electrodes and managed to create conducting paths all over the triboelectric layers. The fabrication of wearable T‐TENGs has to overcome great challenges, such as flexibility, breathability, washability, softness, lightweight, and comfort properties.^[^ ^69^ ^]^ **Figure** **11** indicates the recent rapid progress of CSYs T‐TENGs and it shows that structural design research results are promising pathways towards the next generation of practical textile TENGs.

**Figure 12:**
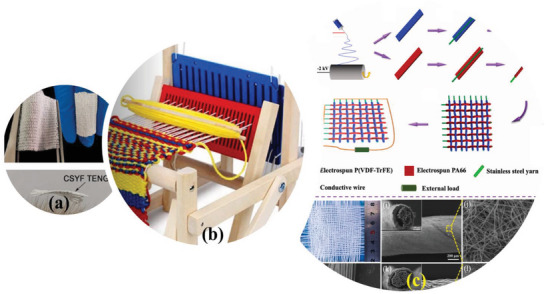
a) Plain weaving pattern samples for CSYs T‐ TENGs, b) portable weaving machine used to male CSY layers, and c) production process of nanofibers‐based core sheath yarns and woven TENG samples, and cross‐sectional SEM view of the corresponding nanofibers. a) Reproduced with permission.^[^ ^55^ ^]^ Copyright 2020, Elsevier. c) Reproduced with permission.^[^ ^79^ ^]^ Copyright 2022, Elsevier.

The structural design of CSYs fabric layers is crucial for the development of the next‐generation‐TENGs, especially for wearable sensing applications. CSYs often need to be braided, woven, or knitted into textiles and are essential techniques for designing layer structures in a variety of fabrics,^[^ ^70^, ^71^ ^]^ Each technique uses a different method to interlace threads or fibers to create a textile structure. Braiding is a technique where three or more threads or fibers are intertwined in a diagonal or spiral pattern. Braids can be flat or round and can be made using a variety of materials, including natural and synthetic fibers, wire, and metal.^[^ ^72^ ^]^ Weaving is a fundamental technique where two sets of yarns the warp, and the weft, are interlaced to create a fabric. The warp yarns run vertically on the loom, while the weft yarns run horizontally. The warp yarns are held under tension on the loom, and the weft yarns are passed over and under the warp yarns using a shuttle.^[^ ^73^ ^]^ In knitting loops of yarn are interlocked to create a fabric, in a variety of patterns, including plain, ribbed, tubular, cardigan ripple, garter, stockinette and etc.^[^ ^74^ ^]^ Each of these techniques has its advantages and disadvantages and is used to create unique textures, patterns, and structures. Weaving,^[^ ^75^ ^]^ knitting^[^ ^76^, ^77^ ^]^ and braiding^[^ ^58^ ^]^ can be adopted depending on the type of surface needed for the triboelectric layers. Most of the woven‐structured T‐TENGs operate in C‐S and L‐S mode and consist of dual type of triboelectric material in single textile layers. Plain weave fabrics are lighter, more breathable, and less prone to deformation.^[^ ^39^ ^]^ Early research on the woven T‐ TENGs started with making of woven electrodes and woven strips of positive and negative triboelectric material separately forming a checker‐like pattern over the electrodes with matching periodicity. The implementation of a positive and negative triboelectric material with core electrodes has significantly improved the performance of the woven T‐TENGs. In weaving the process of designing the fabric structure, is facilitated by the interweaving of warp and weft yarns in the fabric, achieved by using a lifting plan of raising the shafts for every weaving course in a dobby loom. Knitting is using carriers to carry the yarn which is laid over several latch needles to create continuous rows of loops which are interloped between them. There is also 3D woven fabric which have a larger space for movement and sufficient contact‐separation between fibers, so a larger electrical output is expected. **Figure** **12** depicts the plain weaving pattern design for 3D T‐TENG fabrics and it also shows the woven checker‐like pattern over electrodes for energy harvesting applications.^[^ ^78^ ^]^

In section 2 large scale fabrication of CSYs by employing braiding techniques is considered in detail. Several efforts are made to develop the CSY structure by normal yarn winding, twisting, or braiding techniques.^[^ ^80^ ^]^ Liyun Ma et.al investigated a 3D five‐directional braiding structure and assembled a 3D braided TENGs for biomechanical energy harvesting applications. The frequent spatial frame‐column structures formed between the rhombic braided braced frame and the axial core column generate a contact‐separation space, endowing the 3D braided‐TENGs with apprehensions regarding air permeability, pressure sensitivity, power output and compression resilience. Silver‐coated nylon yarn and silicone rubber elastomer based stretchable yarn with built‐in spring‐like spiral winding were also investigated by braiding. An internal core column was inserted into the sheath tube and a spring‐like structure is created in which the braided yarns show higher sensitivity to mechanical stimuli.^[^ ^81^ ^]^ Metallized Silver, Copper, Stainless Steel, Nickel, Graphene‐Carbon plated filaments act as electrodes in most braided CSYs productions.^[^ ^82^ ^]^

The metalized stretchable electrode yarns lead to better performance towards the CSYs braided T‐TENGs output due to the high internal conductivity and higher contact surfaces between the core electrode and the tribo materials. Gao et. al. developed a CSYs based T‐TENGs with a hierarchical architecture of braided CSYs. The device performance is more stable, it produced an output voltage of 174 V, and an average power density of 57 mW/m^2^.^[^ ^30^ ^]^ Another type of hierarchical structure (HS) was explored by Fei Wu et. al by producing a helically structured fiber with Ti_3_C_2_T_x_ as the triboelectric coating material. This distinctive architecture delivers the HS‐TENG with large stretchability (∼ 200% strain), and reasonable electric output performance (52 V, 1.5 µA, 4.2 µW) under compression conditions.^[^ ^31^ ^]^ A team of researchers led by Junyi Zhai developed a stretchable coaxial triboelectric nanogenerator (TENG) yarn. This innovative yarn was fabricated by utilizing a coil spring as the inner support layer, combined with a mechanoluminescent ZnS: Cu/PDMS composite as the outer friction layer.^[^ ^84^ ^]^ The same team also introduced a yarn‐based elastic E‐braid with a kernmantle structure to explore its application potential in high‐impact sports monitoring. This approach overcomes the inherent low elasticity of CSYs‐based electronic devices, opening up new possibilities in the field.^[^ ^85^ ^]^ **Table** **1** describes the most recent research progress on braided CSYs T‐TENG devices and its application towards real time monitoring of various sensing parameters.

**Table 1:** Summary and comparison of different braided CSYs T‐TENG device performance

Sl. No	CSYs Fabrication Techniques	Tribo Material Pairs	Electrode Material	Voltage (V)	Current (µA)	Power Density (mW m^−2^)	Sensing Applications	Ref
01	Spinning/Coating/Braiding	Nylon‐ Polydimethylsiloxane (PDMS)	Silver‐plated Nylon	174	5.1	57	Motion	[30]
02	Winding	Polyurethane (PU)‐ Polyethylene terephthalate (PET)	Ag NW‐fabric/ Ti_3_C_2_Tx	160	8	21	Motion	[31]
03	Braiding	Polyimide (PI)‐Polyurethane (PU)	Silver‐coated Polyamide (PA)	17.5	0.068	–	Strain	[33]
04	Winding	Nylon‐ Silicone	Silver‐coated Nylon	19	0.43	8	Gesture	[81]
05	Braiding/Winding	Skin‐ Polydimethylsiloxane (PDMS)	Silver‐plated Nylon	90	0.75	260	Pattern recognition	[83]

**Figure 13:**
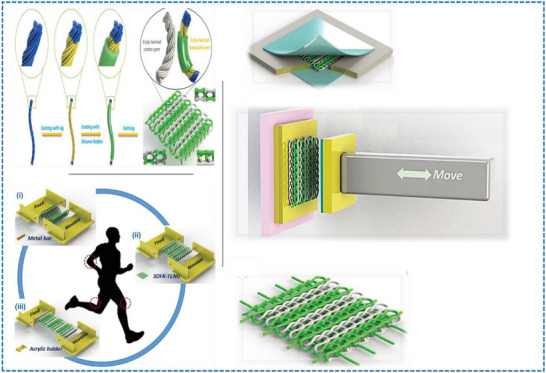
Schematic representation of fabrication of CSYs, knitted CSYs T‐TENG and testing demonstration of 3D knitted CSYs‐TENGs with respect to various bending angles. Reproduced with permission.^[^ ^86^ ^]^ Copyright 2019, Elsevier.

Ronghui Wu et.al examined a 3D braided stretchable hierarchical interlocking fancy‐yarn TENG with deoxyribonucleic acid‐like double‐wing spiral structure. This TENG produced mechanical robustness of 6.9 cNdtex^−1^ and stretchability of 350%.^[^ ^33^ ^]^ **Figure** **13** shows a schematic view of this knitted CSYs T‐TENG and the behaviour of the 3DFIF‐TENG in respect to various bending angles. Zhou et.al extended the stretchable hierarchical approach from micro to nano CSYs. The yarn structure was different from chemical coated yarns; their uniaxially aligned CSYs are made with ordered nanofibers tightly wrapping around the conductive core fibers, which are manufactured via a one‐step conjugate electrospinning technique called double spinning, using a continuous yarning system. Owing to the hierarchical structure of the yarns and their higher surface area, CSYs T‐TENGs exhibit high electrical output performance. Furthermore, based on the micro/nano radial expansion fiber based fabric architecture, their excellent sensing abilities are manifested by making nanofibers based CSYs T‐TENGs for various sensing applications.^[^ ^32^, ^55^ ^]^

**Figure 14:**
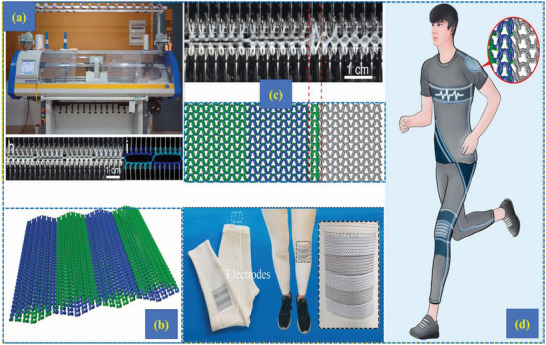
a) Computerized flat knitting machine, b) schematic diagram of knitted T‐TENGs samples, c) design and fabrication of the T‐TENG samples using a flat knitting machine and d) application of knitted T‐TENGs in day today life. Reproduced with permission.^[^ ^96^ ^]^ Copyright 2020, Elsevier.

Liyun Ma et.al fabricated a 3D T‐TENG with a honeycomb structure which is woven by flame‐retardant continuously spun yarns.^[^ ^78^, ^83^ ^]^ The honeycomb weaving structure had benefits in similarity to other weaving structures.^[^ ^87^ ^]^ The same group further fabricated an ultralight Single‐Electrode Triboelectric Yarn (SETY) with helical hybridized nano‐micro core−shell fiber bundles by a facile using double electrospinning. The achieved SETY device exhibits ultra‐lightness (0.33 mg cm^−1^), extra softness, and small size (350.66 µm) in comparison with conventional fabrication techniques.^[^ ^38^ ^]^ Yujue Yang et. al explored the combination of core‐spun method and a coating approach together, and shown that triboelectric materials can be incorporated on the surface of conductive fibers with forming of staple structure with enhanced interfacial properties.^[^ ^63^ ^]^ Guan et.al projected a new kind of woven‐structured triboelectric nanogenerator (WS‐TENG), which encompasses stainless‐steel yarns wrapped around by electrospun polyamide 66 nanofiber and polynanofiber, and generated high output performance (166 V, 8.5 µA and 93 mW/m^2^).^[^ ^79^ ^]^ Mengna Lou et. al also explored various CSYs structures to develop the T‐TENG using low‐cost textile forming techniques, incorporating twisting and weaving. They investigated Nylon and PTFE filaments‐based sheath materials with a helical stainless‐steel yarn‐based core electrode layer. This is a completely all‐fiber structure without using any adhesion and created an exceptional twisted helix structure, which induce numerous forms of deformations during wearable applications.^[^ ^88^ ^]^ Enfang He et. al devised a 3D angle‐interlock woven structured T‐TENG by using a silicone rubber coated Graphene oxide/cotton composite yarn (**Figure** **14**).^[^ ^96^ ^]^

The optimal single yarn demonstrates open‐circuit voltage of 30.8 V and short‐circuit current of 1.1 µA using a constant loading tapping force (∼10 N). The obtained 3D‐ TENG reveals the output power of 225 mW/m^2^ with the area of 5 cm X 7 cm, which was comparable with 2D and 3D double‐layer plain structures.^[^ ^93^ ^]^ **Table** **2** describes the most recent research progress on woven CSYs T‐TENG devices and its application towards real time sensing. Xuejiao Tao et.al improved the wearable TENGs performance by weaving polyurethane (PU) nanofiber CSYs and Si_3_N_4_‐electret‐doped polyvinylidene fluoride (PVDF) nanofiber CSYs into a double‐layer fabric. The electrical output performance of the T‐TENGs is stable with open circuit voltage of 71 V, short‐circuit current of 0.7 l A but it was not having textile material properties for wearable applications.^[^ ^52^ ^]^

**Table 2:** Summary and comparison of different woven CSYs T‐TENG device performance

Sl. No	CSYs Fabrication Techniques	Tribo Material Pairs	Electrode Material	Voltage (V)	Current (µA)	Power Density (mW m^−2^)	Sensing Applications	Ref
01	Cutting into strips	Nylon‐ Polytetrafluoroethylene (PTFE)	Silver (Ag) coated Polyamide (PI)	62.9	1.77	5.43	–	[89]
02	Twisting/ surface treatment	Skin‐ Polydimethylsiloxane (PDMS)	Nickel (Ni) and Copper (Cu), Reduced Graphene Oxide/Carbon nanotubes (rGO/CNT)	60	3	1.4	–	[90]
03	Braiding	PA66 (Nylon)‐ Polytetrafluoroethylene (PTFE)	Silver	40	2	11	–	[91]
04	Twisting	Polyurethane (PU)‐ Polyester	Stainless‐Steel (S‐S)	75	1.2	60	–	[92]
05	Wrapping	Nylon‐ Polytetrafluoroethylene (PTFE)	Stainless Steel (S‐S) monofilament	2.17	0.126	0.0099	–	[88]
06	Dip Coating	Skin‐ Silicone	Graphene Oxide	30.8	1.1	225	Motion	[93]
07	Double Electrospinning	Polyurethane (PU)‐ Si_3_N_4_‐doped PVDF	Copper (Cu)	71	0.7 l	–	–	[52]
08	Electrospinning/Wrapping	Polyamide 66 (PA 66)‐ Polyvinylidene fluoride (PVDF)‐ TrFE	Carbon©	166	8.5	93	Touch	[79]
09	Double Electrospinning	PCL – Polyvinylidene fluoride (PVDF) / Polytetrafluoroethylene (PTFE)	Silver (Ag)/ Nylon	20	2.2	2.2	Humidity/ Pressure	[55]
10	Double Electrospinning	Silica aerogel/ Polyamide‐ Polytetrafluoroethylene (PTFE)	Carbon ©	5V	0.00008	–	Temperature	[32]
11	Double Electrospinning	Skin‐ PAN/ Polyvinylidene fluoride (PVDF)	Silver (Ag)	40.8	0.070	0.033	Pressure	[38]
12	Ring spinning/dip coating	Thermoplastic Polyurethane (TPU) – Polydimethylsiloxane (PDMS)	Silver (Ag)	117	2.3	213	Motion	[63]
13	Melt spinning	Skin‐ Silicon	Gallium‐Indium‐Tin Liquid Alloy	175	15	469	–	[65]

Knitted fabrics are soft, have good wrinkle resistance and breathability, and have greater extensibility and elasticity.^[^ ^58^, ^92^, ^94^, ^95^ ^]^ ShanshanDong et. al described stretchable and comfortable T‐TENG knitted layers, which consist of two knitwear layers that are alternatingly parallel to one another and crossing at the junctions.^[^ ^96^ ^]^ Further Yongyun Mao et.al proposed a round‐tripping‐knitting strategy which is devised to directly knit flexible PTFE yarn around the PDMS/MnO_2_NW coated conductive carbon cloth yarns.^[^ ^97^ ^]^

Even though the output of some of these investigations is high, transferring those high energy outputs to practical wearable applications is still difficult in T‐TENGs due to its high impedance mismatching between source and load. The storing of the T‐TENGs generated energy in the textile itself is one of the pathways to mitigate the charge transfer issues. Mengmeng Liu et.al developed yarn‐based TENGs (Y‐TENGs) with yarn asymmetric supercapacitors (Y‐ASC) for energy‐storage, trying to solve the major storage problem of‐TENGs.^[^ ^90^ ^]^ **Table** **3** describes the most recent research progress on knitted CSYs T‐TENG devices and its application towards real time sensing.

**Table 3:** Summary and comparison of different knitted CSYs T‐TENG device performance

Sl. No	CSYs Fabrication Techniques	Tribo Material Pairs	Electrode Material	Voltage (V)	Current (µA)	Power Density (mW m^−2^)	Sensing Applications	Ref
01	Braiding	PA66 (Nylon)‐ Polytetrafluoroethylene (PTFE)	Silver (Ag)	32	1.9	1.484	–	[98]
02	Dip Coating	Polyamide (PA) /Cotton‐ Silicone Rubber	Silver (Ag)	8	0.003	0.34	Tactile	[86]
03	Twisting/Spinning	Cotton/Nylon‐ Polytetrafluoroethylene (PTFE)	Stainless Steel (S‐S)	7.25	0.016	0.000004	–	[99]
04	Spinning/roll‐to‐roll dip‐coating/ Multiaxial winding	Nylon‐ Polyesters	Graphene	30	1.2	1.03	Motion	[100]
05	Ring/Electrospinning	Polyamide 11 (PA11)/ Zinc Oxide (ZnO) – Polydimethylsiloxane (PDMS) /Polyester	Polycarbonate (PCY)	55	0.5	48.78	–	[101]

## 4 Integration of Charge Extraction and Energy Storage Systems with CSYs T‐TENGs

Miniaturized self‐powered wearable device networks play a vital role in the exponential growth of the modern AI‐assisted Internet of Things (IoT) era, which will provide a significant foundation for smart healthcare, well‐being, and sports monitoring with respect to smart wearable electronic textiles. The embedding of traditional battery backup with wearable sensor networks still poses challenges due to massive scale manufacturing of toxic materials used in battery production, and environmental depletion, with their weight and size are also other important considerations.

TENGs have the potential to replace batteries in wearable IoT smart devices by providing a sustainable and long‐lasting source of power. T‐TENGs do not require a constant supply of new materials to function, unlike batteries, which require frequent replacement and disposal. Additionally, T‐TENGs can be integrated into the design of smart devices, making them more compact and lightweight. Despite good progress however, there are some challenges reported here, that need to be addressed before T‐TENGs can fully replace batteries in wearable IoT devices. An added advantage of T‐TENGs is, that they can be also used as sensors by being able to detect and provide multiple information from the user and its environment. Hence T‐ TENGs have the capabilities of acting as ion‐sensitive electrochemical sensors,^[^ ^102^, ^103^, ^104^ ^]^ displacement, acceleration sensors,^[^ ^105^ ^]^ humidity sensors,^[^ ^106^ ^]^ temperature sensors,^[^ ^107^, ^108^ ^]^ biomedical and other sensors.^[^ ^109^, ^110^, ^111^, ^112^ ^]^ However, to date, as already discussed T‐TENGs are not capable of producing high power densities that are equivalent to those in batteries, which limits their use in wearable self‐powered applications. Moreover, T‐TENGs require consistent and predictable mechanical inputs to generate constant output, which may not always be available in real‐time wearable conditions.

**Figure 15:**
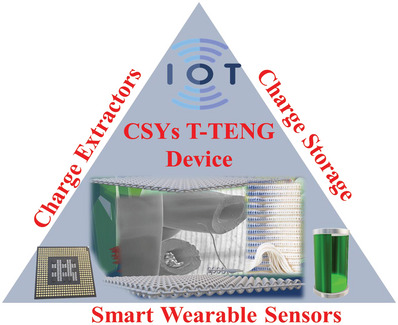
Schematic illustrations of IoT enabled CSYs T‐TENGs for smart wearable applications.

Therefore, although current research focus is on T‐TENGs by combining their ability of power generation and sensing capabilities, to enable the use of CSYs T‐TENGs for practical applications, researchers also need to investigate the most appropriate self‐charge extractor circuits, charge management and regulator circuits, as well as suitable energy storage devices (**Figure** **15**). These requirements have been achieved in several traditional TENGs; however, the additional rectifier bridges and transistor‐based switch circuits needed for CSYs greatly increase the complexity of the entire device and goes against the trend of miniaturized wearable smart applications. The high‐voltage and low‐current output characteristics of a T‐TENGs are due to the high internal impedance which makes it difficult to directly power smart wearable sensing devices. TENGs have high output impedance, generally in the range of several MΩ. But the input impedance of any common DC load device is only several Ω and impedance mismatch occurs.

**Figure 16:**
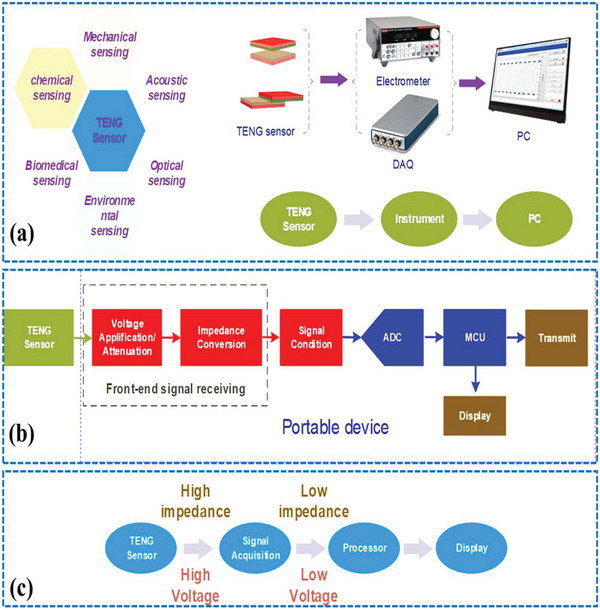
a) Standard measuring equipment for TENGs characterization and possible sensing application areas, b) block of diagram of signal processing units of TENGs devices and c) shows the necessity of the signal processing circuit for TENG sensors. Reproduced with permission.^[^ ^113^ ^]^ Copyright 2021, Elsevier.

In addition to that, energy harvesting, storing and distributions are not synchronized, which necessitates effective electronic solutions. Self‐charge extractor circuits play an important role in energy harvesting systems and are used to extract the electrical energy generated by the TENGs and convert it into a usable form for powering electronic devices.^[^ ^114^ ^]^ It will help to maximize the efficiency of energy conversion from the traditional TENG which is equally applicable in the CSYs‐TENGs. **Figure** **16** describes the standard method of measuring setup and power management circuits (PMCs) for CSYs T‐TENGs.

**Figure 17:**
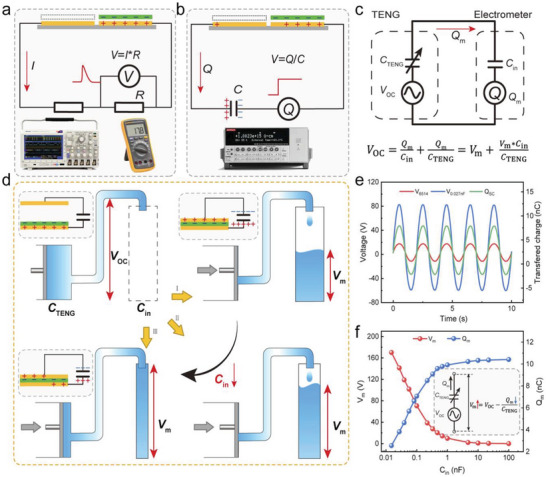
a–b) principles of measuring the output voltage of TENG with respect to the resistance and capacitance based apparatuses, c) equivalent circuit of an electrometer measuring the output voltage of TENG, d) schematic illustration of the outcome of the instrument's internal capacitance on the measured voltage, e) waveform comparison of measured voltage and short‐circuit transferred charge measured by a electrometer and an improved electrometer with the *C*_in_ value of 0.027 nF, f) dissimilarity of measured voltage and transferred charge with the increase of *C*_in_. Reproduced with permission.^[^ ^115^ ^]^ Copyright 2022, Elsevier.

The calibration set up for TENG measurement is very crucial for energy harvesting and sensor development, and it directly affects the output performance and sensitivity of the device. The mechanical properties of the CSYs T‐TENGs differ. These are the contact area, the pressure acting on the surface, the roughness of the surface, the environmental conditions (temperature, humidity, and air flow), and the method of measurement, and all of these affect the overall output signal. The measurement system setup, including the grounding, shielding, and wiring, can affect the noise level, interference, and signal‐to‐noise ratio of the measurement. **Figure** **17** illustrates the reasons why the measurement method and the internal impedance of a voltmeter can significantly affect the voltage measurement outcomes. The equivalent circuit of an electrometer measuring the output voltage of TENG and the correlation between V_m_/V_OC_ and C_in_/C_TENG_ are described in detail (Figure 17c).^[^ ^115^ ^]^

Only few investigations are available on the charge excitations, power management, storage, measurement setups, and sensing abilities of CSYs T‐TENGs compared with traditional TENGs. More research is needed to mitigate current difficulties. Chao Ye et al integrated a SC‐TENG yarn into an IntelliSense system to recognize and control various electronic and electrical systems. IntelliSense fabrics that used to sense transient mechanical stimuli, are extensively predictable in flexible and wearable electronics. They demonstrated further the device application potential towards wearable energy supply.^[^ ^116^ ^]^ Recent self‐charge excitation methods are highly focused on traditional TENG to improve the charge transfer capabilities.^[^ ^117^, ^118^, ^119^, ^120^ ^]^ The elementary power generation mechanism is based on the charge transfer between two groups of capacitors. Consequently, if the external capacitor cluster can comprehend the automatic switch from the parallel to serial connection during the contact and separation process of TENG devices, the doubled charges from the parallel‐connected capacitors could feed back to the TENG component and thus implement the function of self‐excitation.^[^ ^114^ ^]^ Wenlin Liu et.al designed self‐excitation circuits for traditional TENGs. Circuits are built in a voltage multiplier circuits (VMC) group which consists of three rectifier diodes and two ceramic capacitors to produce the self‐charge excitation mechanism. Various types of trigger switches are introduced to modulate the output performances of TENGs, which include the MOS‐FET based switches in these PMCs, but these are composed of active electronic components, which require additional power back up to drive the switches. The active electronic components increase the complexity, size, and manufacturing cost of the PMCs system especially in textile‐based platforms. There is great demand for developing unique impedance matching circuits, and which should be from passive electronic components, including an inductor, a diode, and a capacitor and this can solve the complexity of the circuits and the need of using external power sources.^[^ ^121^ ^]^

In addition to the charge extracting circuits, researchers are trying to develop yarn/fabric‐based energy storage systems. Textile fabric is an ideal substrate for wearable energy‐storage devices since it is soft, mechanically flexible, and highly porous with abundant surfaces for absorbing electrochemically active materials. Mengmeng Liu et.al developed yarn‐based TENGs (Y‐TENGs) with yarn asymmetric supercapacitors (Y‐ASC) as the energy‐storage unit. It consists of a negative yarn electrode with hydrothermally self‐assembled rGO/CNT coating and a positive electrode of electroplated Ni‐Co bimetallic oxyhydroxide (NiCo BOH) coating.^[^ ^90^ ^]^ Highly conductive 1D yarns were achieved by electroless deposition (ELD) of Ni and sequential electrodeposition of Cu coatings on polyester yarns and applied as current collectors in Y‐ASCs.

**Figure 18:**
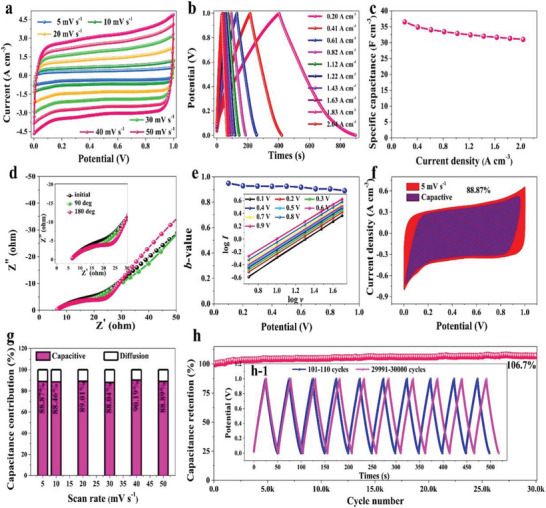
a)Current versus voltage (CV), b) GCD, c) volumetric specific capacity, d) EISs at different bending angles. e) The b‐values for CC@MnO_2_NW electrode, f) capacitive charge storage contributions at scan rates for CC@MnO_2_NW electrode, g) area normalized contribution percentage of capacitive capacitance at different scan rates, h) charge–discharge cycling stability for CC@MnO_2_NW electrode. Reproduced with permission.^[^ ^97^ ^]^ Copyright 2022, Elsevier.

The active material of the positive electrode is Ni‐Co bimetallic oxyhydroxide, which is electrodeposited on the surface of Cu; the rGO/CNT hybrid film on the negative electrode is originated by a spontaneous redox reaction between the Cu and GO/CNT suspensions. Further an asymmetric supercapacitor was built with the negative and positive yarn electrodes and separated by a solid poly (PVA)/KOH gel electrolytes.^[^ ^90^ ^]^ **Figure** **18** exhibits the electrochemical performance (CV, GCD, volumetric specific capacity) of CC@MnO_2_NW electrode. The superior power generation performance of the T‐TENGs array also obtained by modulating the dielectric permittivity of the materials, for example the PDMS/MnO_2_NW elastomer by mass loading of MnO_2_NW.^[^ ^97^ ^]^

The CSYs T‐TENGs can not only convert mechanical energy into electric energy, but also act as a sensing device. T‐TENGs based sensors detect a wide range of physical stimuli, including mechanical, thermal, and chemical, with high sensitivity. The combining of the dielectric material properties of the CSYs with TENG can be utilized to monitor human body activities. The basic sensing capabilities used in the TENG comes from two objects that are in contact and are separated; and so electric signals in TENGs are produced by the applied pressure and frequency. Here the intensity and number of peaks, as well as the temporal sequence characteristics, are notable signs to recognize the sensing signals caused by different external stimuli.

**Figure 19:**
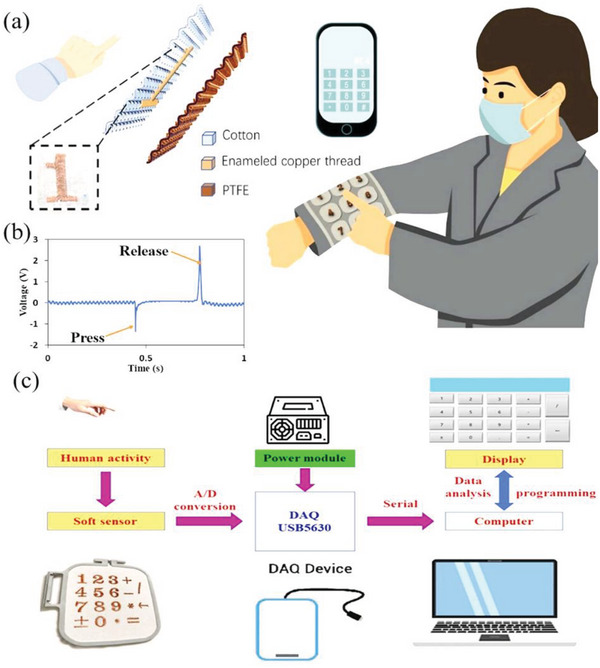
a) Example of a CSYs TENGs based embroidery numeric keypad, b) Output voltage variations during finger pressing and releasing c) a fully fabric‐based numeric keypad and corresponding electronic system. Reproduced with permission.^[^ ^122^ ^]^ Copyright, Elsevier.

However, the pattern of triboelectric signals is difficult to be distinguished by humans in many cases, so it is challenging to set provisional decision algorithms. To improve the sensing accuracy, Machine Learning (ML) and AI models are employed, integrating the ML assisted fabrics with the Internet of Things (IoT) platforms for achieving real‐time monitoring.^[^ ^116^ ^]^ The consequence of the mode of motion on the frictional electricity signal is replicated in the change of the peak value of open‐circuit voltage and conveys these variations in frequency and overall signal patterns. This variation is used to analyse the motion intensity and motion patterns^[^ ^122^ ^]^ and acts as a sensor. Recently electromagnetic shielded yarns are progressively advancing towards intelligence TENG stimuli, this is a pioneering method toward self‐powered human‐interactive and functional protective textiles for the next‐generation of intelligent sensors.^[^ ^123^ ^]^ The investigation of a triboelectric embroidery numeric keypad working with a terminal device is illustrated in the **Figure** **19**.

**Figure 20:**
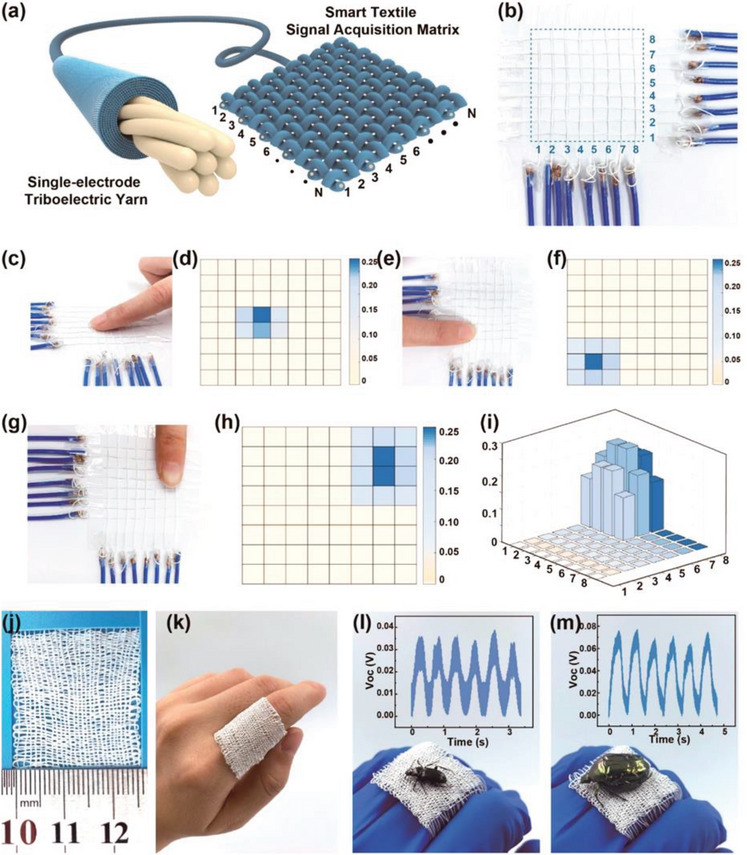
a,b) 8 × 8 pixel sensing fabrics, c,e,g) multiple sensing fabric, d,f,h) corresponding voltage signals during finger touch, i) 3D representation of the output signals, j,k) high‐density plain fabrics, i,m) sensitivity measurement of the fabric during the two different insect specimen touches. Reproduced with permission.^[^ ^38^ ^]^ Copyright, 2020, American Chemical Society.

Plantar pressure distribution is widely identified in human disease diagnosis, biomechanics, and gait analysis, which leads to smart CSYs T‐TENG socks capable of detecting the distribution of plantar pressure. These smart CSYs T‐TENG socks are composed of three main parts: smart textile fabric(s), signal processing circuit and software. The voltage signals are further converted into digital signals through an analog to digital converter and trigger the micro controller unit for data interpretation about plantar pressure. In addition to smart footwear systems, human motion monitoring (heart rate, breathability, motion) and pattern recognition carpets are also reported in CSYs‐T‐TENGs.^[^ ^83^ ^]^ **Figure** **20** shows smart textiles for biomechanical touch sensing using CSYs T‐TENG structures. Voltage signals are captured and transmitted through the IoT enabled wireless modules, controlled by miniaturized micro controllers enabled with Wi‐Fi, Bluetooth, or RF modules. The data processing units at the same time determine, evaluate, and estimate the obtained wireless signals to show the step number, average velocity, and exercise intensity of the user, using a software output interface.^[^ ^30^, ^38^, ^60^, ^86^ ^]^ Aifang Yu et al. conducted research on T‐TENGs by creating a pressure‐sensitive carpet for self‐powered fall detection. This carpet demonstrated good electrical response and exhibited a high‐pressure sensitivity of 0.07 kPa^−1^ within the range of 0.8 to 11.8 kPa. Additionally, the T‐TENGs exhibited a fast response time of less than 28 ms, enabling quick recognition of falling actions. Essentially, the system could accurately judge the falling action and send signals without interfering with normal daily activities, thus maintaining a seamless living experience.^[^ ^124^ ^]^ Furthermore, the research team successfully developed a self‐powered trampoline sport dual‐mode sensing system utilizing E‐Braids. During trampoline activities, the E‐braid generates an output signal that is transmitted to a data acquisition module. Real‐time sensing signals are captured and relayed back to the computer. Through signal processing, the system generates visualized results, offering significant insights into the dynamics of trampoline sports.^[^ ^85^ ^]^

**Figure 21:**
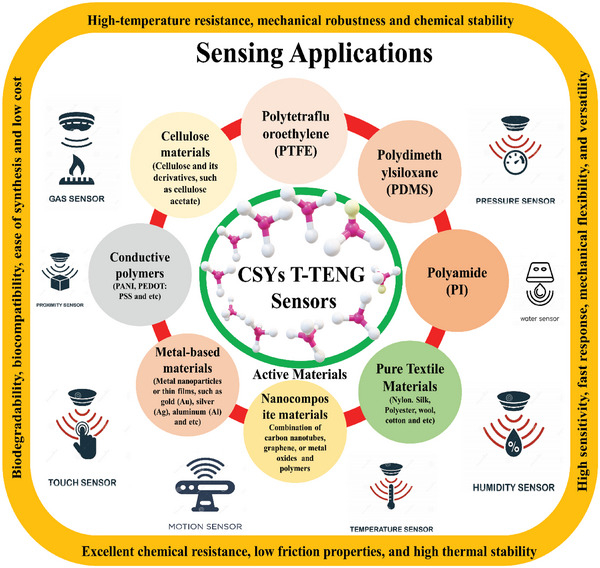
A schematic illustrations of triboelectric materials used for sensor development, and their advantages and applications.

Triboelectric materials (Polydimethylsiloxane (PDMS), Polytetrafluoroethylene (PTFE), Polyethylene (PE), Polypropylene (PP), Polyimide (PI), cellulose‐based materials, conductive polymers, metal‐based materials, nanocomposite materials, and hybrid materials)^[^ ^125^, ^126^ ^]^ are also frequently used in various sensing applications.^[^ ^127^ ^]^ The pressure, strain, touch, motion, pattern recognition, tactile, temperature, humidity, chemical, and biosensors can be monitored and measured in CSYs T‐ TENG using tribo materials and hence we can call “CSYs T‐TENGs as sensors”. **Figure** **21** illustrates the various triboelectric materials used for multivariant sensor developments, and their applications and advantages. These sensors work on different sensing principles ranging from capacitance, resistance, inductance impedance, electrochemical and etc, and are used for wider range of applications such as wearable electronics, robotics, structural health monitoring, touch screens, medical devices, environmental monitoring, industrial safety, healthcare applications, medical diagnostics, point‐of‐care testing, and in biological systems. Current research needs to do a continuous investigation of new fabrication techniques, functionalization methods, and integration approaches to enhance the performance and expand the capabilities of these sensors within the TENGs framework.

## 5 Challenges and Prospects

The CSYs T‐TENGs have attracted substantial attention by the research community due to their unique features, such as electrode‐enabled reliable output performance, low cost, and broad applicability.

**Figure 22:**
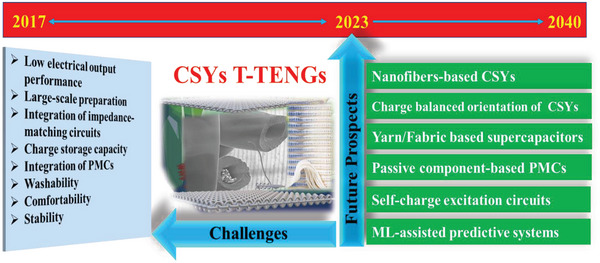
Schematic illustrations of the current challenges and prospects of CSYs T‐TENG considering a time scale window; 2017 to 2023.

Nonetheless, like any emerging technology, CSYs T‐TENGs face numerous challenges that must be addressed to fully realize their potential in real‐time wearable self‐powered sensing applications. Emphasizing the challenges and prospects of CSYs T‐TENGs is important. **Figure** **22** elaborates on the current challenges and future scope considering a time scale window of 2017 to 2023.

### 5.1 Large, Scale Preparation of CSYs

Preparation is a crucial part for the development of CSYs with appropriate electrodes and trip materials. In CSYs, the conducting core provides strength and stability, while the nano/microfibers wrapped around it gives the yarn desirable properties such as softness, warmth, and elasticity for the whole yarn. Finding a suitable highly conducting core material that meets these criteria can be challenging and maintaining uniformity of the dielectric/tribo layer throughout the yarn is also an issue for large‐scale fabrication. The process of wrapping the fibers around the core material requires precision and consistency, however, the chemical coating and the electrospinning methods are complicated and time‐consuming procedures, because any variation in fiber tension or wrapping angle can result in irregularities in the yarn structure which will affect the TENG performance. Achieving uniform fiber wrapping at a large scale requires specialized equipment such as a very precise and stable electrospinning unit with a high voltage precise power source. In many cases, CSYs are made by blending different types of fibers to achieve specific properties. The blending process requires careful consideration of the characteristics of each fiber, such as length, fineness, strength, and durability. Achieving consistent blending at a large scale can be difficult and requires sophisticated blending or coating equipment. CSYs from the laboratory to larger scale commercialization has been attempted, and some progress has been made, unfortunately, CSYs are more expensive to produce than conventional yarns due to the additional materials costs and multiple processing steps involved. Finding an industrial‐compatible, low‐production cost, green, and sustainable fabrication method is a direction that still needs to be discovered.

### 5.2 Low Electrical Output Performance for Real Time Applications

The output performance of CSYs T‐TENGs is affected by several factors that present significant challenges, especially when it comes to real‐time applications. The selection of materials for the core and outer layers is crucial for achieving high electrical output performance. These materials must have a large difference in triboelectric properties to generate a high charge density, but they must also be mechanically stable to withstand the repeated deformation and friction required to generate continuous electrical energy during use. The surface structure of the CSYs T‐TENG affects its electrical output performance and maintaining uniform surface properties is currently difficulty to achieve during fabrication. The outer surface layers must be thin and flexible to maintain guarded contact with the core material and the opposite layer to maximize the triboelectric effect. The manufacturing process must ensure that the core and outer layers are precisely aligned and tightly bound to avoid any separation or delamination that can reduce the electrical output. The process must also minimize any contamination, damage, or inconsistency to the surface of the materials, which can affect their triboelectric properties. Environmental factors such as humidity, temperature, and air pressure can also significantly affect their electrical output performance. The other important aspects with respect to the use of CSYs TENG are load and source impedance matching. The impedance matching circuits must be carefully selected and optimized to ensure that the CSY T‐TENG operates at its maximum power output.^[^ ^128^, ^129^ ^]^

### 5.3 Low Charge Storage Capacity and Unstable Output Performance of CSYs T‐TENG

The storing of energy generated from the CSYs T‐TENG is very crucial for practical applications. However, achieving high charge storage capacity and stable output is challenging due to several factors. The materials must have a large difference in their triboelectric properties to generate high charge density, but they must also have a low leakage current to maintain charge storage capacity over time. Additionally, the design of the electrodes is essential for achieving high charge storage capacity and stable output. The electrodes must be designed to minimize resistance per meter, maximize contact area, and avoid surface defects or contaminants that can reduce the charge storage capacity or affect the output stability of the CSYs T‐TENG. The dissipation of charge stored over time is a significant challenge for achieving high charge storage capacity and stable output. Charge dissipation can be due to various factors, including current leakage, surface defects, and environmental factors such as humidity and temperature. Achieving high charge storage capacity and stable output involves several challenges related to material selection, electrode design, yarn structure, operating conditions, and charge dissipation. Addressing these issues requires careful design and optimization of the CSYs TENG, and a deep insight into the underlying physics of the triboelectric effect and charge storage mechanisms. The development of flexible, bendable, and miniaturized passive components‐based power management circuits and supercapacitors in the TENG device platform are essential for successful wearable sensing applications.

### 5.4 Comfort, Washability, UV‐Protective, Radiative Cooling, and Antibacterial Properties of the CSYs T‐TENGs

In future CSYs ‐T‐TENG fabrications, comfortability and washability are crucial criteria for wearable garments. Incorporating UV‐protective, radiative cooling, and antibacterial properties into CSYs will significantly enhance their suitability for medical and industrial safety applications. The selection of materials for the core and outer layers is pivotal in achieving washability and comfortability. These materials should withstand washing and drying while retaining their triboelectric properties and mechanical stability. Additionally, they must be soft, flexible, and tightly bound to ensure wearability and comfort, preventing separation or delamination due to wear and tear, and washing. Regular washing and drying cycles can lead to a reduction in triboelectric properties, affecting electrical output. Incorporating UV‐protective, radiative cooling, and antibacterial properties into CSYs presents additional challenges such as material compatibility, optimal additive selection, manufacturing techniques, performance trade‐offs, and long‐term effectiveness. A multifunctional approach has been developed, utilizing a chemical modification method to achieve T‐TENGs with outdoor radiative cooling, antibacterial, and UV‐protective properties.^[^ ^130^ ^]^ This approach combines conductive stainless‐steel wires (SS) as electrodes, anti‐UV cotton yarns with antibacterial properties (UV/OM‐CY), and polyethylene (PE) yarns twined around the core SS fibers (PE/UV/OM‐CY). These CSYs demonstrate antibacterial activity against Escherichia coli and Staphylococcus aureus. However, most reported materials (Table 1, 2, 3) lack pure textile characteristics and exhibit lower washability, comfortability, UV protection, radiative cooling, and antibacterial properties compared to conventional textiles.

### 5.5 Integration Difficulties of CSYs‐T‐TENGs with Power Management Circuits

Integrating CSYs‐T‐TENGs with power management circuits presents several limitations due to their variable electrical characteristics, i.e., variable capacitance and high output impedance. Their electrical output is non‐linear, and the output voltage and current vary depending on the mechanical loading, i.e., force and frequency. This non‐linearity can complicate the design and optimization of power management circuits, which typically require a constant or predictable input voltage and current. And because the output voltage and current of CSYs T‐TENGs are typically low, the management of the electrical energy generated is crucial but difficult. Therefore, the power management circuits must be designed to efficiently step up or down the voltage and current levels to meet the requirements of the user application conditions. The load connected to the CSYs‐T‐TENGs is variable, which can affect the output voltage and current. The power management circuit must be designed to adjust the varying load and maintain a stable output. Further than that the power management circuit can introduce power losses, which can reduce the overall efficiency of the system. So, these circuits must be designed to minimize power losses and maximize the power transfer efficiency. Finally, the size and integration of the power management circuits can also be challenging. The circuits must be very small, compact, and unobtrusive and must be integrated with the CSYs‐T‐TENGs to minimize the overall size and weight of the system. This can require advanced circuit printing, packaging and integration techniques, which can add to the complexity and cost of the system.^[^ ^129^, ^131^ ^]^

### 5.6 Low Sensitivity of the CSYs‐ T‐TENGs Based Smart Sensors

CSYs T‐TENGs have the potential for use in smart sensing applications, such as wearable sensors, smart textiles, and environmental monitoring systems. However, there are several difficulties related to their performance and reliability in use. The sensitivity and reproducibility of the sensing performance of CSYs T‐TENGs can be affected by various factors, such as temperature, humidity, and mechanical deformation. These factors can cause changes in the triboelectric properties of the materials, leading to variations in the electrical output of the device and to the calibration process of the sensors. To improve sensitivity, they must be designed to minimize the effects of these factors and maximize the signal‐to‐noise ratio of the electrical output. The selectivity and stability of CSYs T‐TENGs as sensors is also critical for specific applications as they must be designed to respond selectively to specific stimuli, such as mechanical deformation, pressure, or temperature while rejecting unwanted stimuli that can interfere with the measurements, and although this can be mitigated in software it can be an added difficulty. The electrical output of CSYs T‐TENGs can also be affected by manufacturing variations and environmental factors, which can lead to measurement errors. The device must be calibrated to account for these factors and ensure accurate and reliable measurement. The electrical output of CSYs T‐TENGs can be noisy and non‐linear, due to the textile structure, making signal processing needing advanced processing techniques, such as filtering, amplification, which are required to extract the desired electrical output from the device. Hence future research needs to focus on developing CSYs T‐TENG‐based sensors with high efficacy for a variety of sensing applications such as; ion‐sensitive electrochemical, displacement, acceleration, humidity and temperature.^[^ ^109^, ^110^, ^111^, ^112^ ^]^ If this is achieved, it will enable the use of CSYs T‐TENGs with total autonomy combining energy and sensing from the same device in modern IoT‐enabled smart wearable devices and garments.

### 5.7 Design Complexity of Self‐Charge Excitations and Impedance Matching Circuits

Self‐charge excitations and impedance matching circuits are indispensable components of the CSYs‐T TENGs device. Self‐charge excitations occur when the charged surface of the triboelectric material generates an electric field that induces charges in the opposite direction. This can reduce the overall efficiency of the CSYs T‐TENG by reducing the net charge transferred between the two materials. To overcome this challenge, the CSYs T‐TENG must be designed to minimize self‐charge excitations by optimizing the material properties and the design of the device. Impedance matching is critical for maximizing power transfer efficiency between the CSYs T‐TENG and external load. The impedance of the load should be matched with the output impedance of the CSYs T‐TENG to ensure maximum power transfer. However, impedance matching can be challenging because the output impedance of the CSYs T‐TENG is highly nonlinear and dependent on the mechanical input, i.e., force and frequency. To address this challenge, impedance matching circuits must be designed to adapt to the changing output impedance of the CSYs T‐TENGs. To minimize the effects of noise, the CSYs T‐TENG must be designed with low‐noise materials and components, and the circuitry must be shielded from external interference. The frequency response of the CSYs T‐TENGs is critical for many applications, such as energy harvesting and sensing. However, its frequency response can be limited by the mechanical resonance of the device and the response time of the electronic circuit board. To overcome this challenge, the CSYs T‐TENG must be designed with optimized mechanical properties and the electronic printed circuit must be designed to have fast response time. The self‐charge excitations and impedance matching circuits present several challenges that affect the performance of CSYs‐T‐TENGs, such as reducing the efficiency of the device, adapting to the changing output impedance, minimizing noise, and optimizing frequency response. Focusing on these challenges requires careful design and optimization of the CSYs T‐TENGs and their electronic circuit.

### 5.8 Integration Complexity of CSYs T‐TENG with Internet of Things (IoT)

IoT applications require wireless communication platforms for data transmission, which can be difficult to implement with CSYs T‐ TENGs, because they require specialized wireless communication modules, that should be more adaptable to the fabrics, i.e., being flexible and bendable. The wireless data transmission modules can increase the size of the system and power consumption of the device. Additionally, the wireless communication range may be limited due to the low power output of the CSYs T‐TENGs. Integrating CSYs T‐ TENG with IoT applications is also difficult due to the requirement for specialized electronic circuits, including impedance matching and power management circuits, as already discussed. Sophisticated signal processing is required to extract the desired information from the electrical output, as also discussed, which can increase the complexity of the electronic circuits in the garments.

The emergence of wearable devices has revolutionized the way we interact with the IoT and AI which is now embedded in all these systems. Wearable smart textile devices are not only fashionable but also functional, and they have found applications in several fields, including healthcare, sports, entertainment, and are changing our lives. The CSYs T‐TENG has the potential to transform the wearable textile device industry due to its ability to generate power for wearable devices and their sensing ability from the same material and at the same time. This is particularly important for applications where traditional batteries are not practical to use or where frequent battery replacement is inconvenient. Smart sensing involves the use of sensors to collect data about the wearer's environment such as temperature, humidity, pressure, and motion and about our health and well‐being by means of vital body parameters such as movement, acceleration, heart rate, ECG, EEG, body temperature, skin resistivity, etc., so the comfortability of wearable devices is crucial for their widespread adoption. Additionally, the CSYs T‐TENGs need to be designed to be washable, ensuring that the wearable device remains hygienic, as already discussed. The CSYs T‐TENG can be integrated with IoT‐enabled wearable devices, enabling wireless communication and data transfer. This allows for remote monitoring and control of the wearable device, enhancing its functionality.

It is anticipated that prospective investigations on nanoscale yarns will lead to tremendous transformation in the textile TENG energy sector and here we listed in a road map the challenges and how they may be overcome, to realize the ultra‐high output of future CSYs T‐TENGs. This roadmap supports the development of efficient, effective, and practically possible self‐powered smart wearable devices. In summary.

- The top‐down transformations of CSYs to core spun/sheath fibers (CSFs) will be the ultimate success in the Textile TENGs, which will realise thousands of CSF T‐TENGs in a single yarn and lead to the desired ultra‐high output performance.
- Development of ultra‐high conductive flexible electrode, i.e., from MXenes/ Graphene‐Carbon yarns will lead to faster charge absorption from the tribo layers.
- Expansion of inbuilt yarn/fabric‐specific supercapacitors/transistors will drive the power management circuits to become more adaptable to the textile platforms.
- The realization of biocompatible/biodegradable materials‐based microlevel printable electronic circuits within yarns or fabrics is a significant development. These circuits encompass components for well‐designed impedance matching circuits, self‐excitation circuits, and voltage regulators at the micro level. This breakthrough not only aids in the reduction of electronic waste but also facilitates the creation of wearable devices that seamlessly blend into the environment, leaving behind no lasting ecological footprint.
- Investigation of pure textile‐based tribo materials will enhance washability and comfortability of the CSYs T‐TENGs during wear.
- Development of a more realistic CSYs T‐TENGs testing setup is crucial to measure and calibrate the self‐powered sensing devices. The testing setup should measure the contact separation and lateral movement effects with respect to force, frequency, tensile, shear, and compression imposed on the materials. The measurement of the relationship between structure and material mechanics in relation the T‐TENG output is very crucial, and its ultimate fabrication into a whole wearable garment design for practical uses.
- The optimisation of the wearable garment design to fit the T‐TENG energy and sensing requirements without any discount in aesthetics and wearing comfort need to also be considered, for consumer acceptance.

CSYs or CSFs fabric architectures can further be optimized to achieve even higher output and hence fulfil the practical requirements of fabric TENGs for wearable end uses such as medical, sports, well‐being, and beyond. The highlight of this review analysis is supported by current efforts in developing of real fabrics for achieving the realization that the whole garment and not only small parts of fabric can become autonomous T‐TENGs without compromising on the desirable flexibility, wearability, comfort and aesthetics of the material. In a nutshell the potential of CSYs T‐TENG for wearable applications is significant, and with further research and development, CSYs T‐ TENG could become a ubiquitous source of power and sensing for wearable devices and sensing platforms.

## 6 Conclusion

This review paper has provided a comprehensive, critical, and timely overview of the most recent research progress and current challenges in the development of CSYs T‐TENGs. The paper has discussed the fundamental principles of T‐TENGs, it focused on the unique advantages of CSYs T‐TENGs, it analysed the various fabrication methods and materials used in CSYs and CSYs T‐TENGs and highlighted the recent advancements in their integration with charge excitation, energy storage, power management and IoT‐enabled smart sensing applications. The potential end uses of wearable CSYs T‐TENGs are vast and varied, from environmental monitoring to healthcare conditions, and beyond. Despite significant research efforts however challenges remain, such as optimizing the performance and stability of CSYs T‐TENGs and addressing issues related to scalability, reliability, output performance, power management, energy storing, sensing ability and commercialization. Overall, this review paper highlights the tremendous progress made in the development of CSYs T‐TENGs and the challenges posed for its ultimate practical adaptation. The latter part of the review provides a research road map discussion in how these challenges can be overcome. It is expected that continued research and development in this field will lead to the real time implementation of wearable CSYs T‐TENGs into entire fabrics, fulfilling the requirements of sustainable and energy‐efficient technologies for wearable applications.

## Conflict of Interest

The authors declare no conflict of interest.
